# Pregnant Women’s Experiences during Hurricane Maria: Impact, Personal Meaning, and Health Care Needs

**DOI:** 10.3390/ijerph18168541

**Published:** 2021-08-12

**Authors:** Georgina Silva-Suarez, Silvia E. Rabionet, Carmen D. Zorrilla, Hulda Perez-Menendez, Solaritza Rivera-Leon

**Affiliations:** 1 Department of Sociobehavioral and Administrative Pharmacy, Nova Southeastern University, San Juan, PR 00926, USA; 2 Department of Sociobehavioral and Administrative Pharmacy, Nova Southeastern University, Davie, FL 33328, USA; rabionet@nova.edu; 3 School of Public Health, Medical Sciences Campus, University of Puerto Rico, San Juan, PR 00936, USA; hulda.perez@upr.edu (H.P.-M.); Solaritza.rivera@upr.edu (S.R.-L.); 4 School of Medicine, Medical Sciences Campus, University of Puerto Rico, San Juan, PR 00936, USA; carmen.zorrilla@upr.edu

**Keywords:** hurricane, natural disasters, pregnant women, prenatal stress, pregnancy

## Abstract

During a disaster, pregnant women are considered among the most vulnerable. Background: On 20 September 2017, the Caribbean was hit by a category 4 hurricane. The purpose of the study was to explore the impact on pregnant women during and after the hurricane regarding access to health care, social services, and support systems. Methods: In-depth interviews were conducted to 10 women that were pregnant during the event. Qualitative inquiry based on the Interpretative Phenomenological Analysis framework was used to interpret the narratives. Results: Five major themes emerged: meaning of living through a disaster, fear, the dual burden of protecting themselves and their unborn baby, disruption in health care, and coping mechanisms. Despite the negative feelings, most participants experienced positive transformations. They narrated how they stayed calm and coped in order to protect their pregnancy. Their overall evaluation of the healthcare system was positive. The support of friends and family was crucial pre and post-disaster. Conclusions: The interviews provided a wealth of firsthand information of women experiencing a natural disaster while pregnant. The findings underscore the need to incorporate emotional support in the preparedness and response plans for pregnant women. Educating, empowering, and incorporating families and communities is vital in these efforts.

## 1. Introduction

Women, in general, are more likely to be affected by natural disasters than men, mainly due to their role in our society [1,2]. Women are often the ones responsible for providing shelter and covering the basic needs of their families [1,2]. Studies have reported that women’s quality of life can be negatively affected after a disaster [2]. Other studies have reported adverse effects on women’s mental health, reporting more fear and stress and post-traumatic stress disorder (PTSD) [3,4]. 

During a disaster, pregnant women and their newborns are considered among the most vulnerable [5]; mainly because the experiences of pregnant women will affect not only their health but also their unborn babies [6]. Natural disasters can be a stressful situation for the individuals who experience them. Studies have shown that prenatal maternal stress could adversely affect fetal development, which could impact childhood and adulthood [7,8]. Challenges to pregnant women’s health in times of emergency may include restricted access to healthcare, psychosocial and physiological stressors, and adverse birth outcomes [9]. In addition, women in labor cannot wait until the services are restored to deliver their babies. In Puerto Rico, four days after Hurricane Maria, only three major hospitals were operating [10].

On 20 September 2017, the Caribbean was hit by the force of category four Hurricane Maria. Maria has been cataloged as the most destructive and costliest hurricane to hit Puerto Rico [11]. The hurricane crossed Puerto Rico from the southeast to the northwest, and the damages were unprecedented. It destroyed the power system and caused extensive damages to buildings, homes, and roads [11]. Every infrastructure on the island was affected, somehow, and hospitals were not the exception. Most of the island’s hospitals were left without electricity, and 16 days after the hurricane, only 25 out of 78 hospitals were working [10].

The purpose of the study was to explore the impact, personal meaning, and interpretation of pregnant women during and after Hurricane Maria regarding access (or lack of) to health care, medical and social services, and support systems.

## 2. Materials and Methods

This qualitative study used the Interpretative Phenomenological Analysis (IPA) framework. IPA explores how persons make sense of their everyday life experiences [12]. It is used to examine an experience or phenomenon; asking the participants to explain such a phenomenon “the way it occurs and in their own terms” [12]. The IPA approach allowed researchers to gather the vivid memory of participants’ lived experiences as pregnant women during Hurricane Maria in Puerto Rico, in addition to the meaning they ascribed to those experiences. By recalling the event, participants are prompted to share not only recollection of events but persistent memories and feelings about their experiences.

Study participants were recruited using purposeful sampling. This technic seeks to identify individuals that are especially knowledgeable about or experienced with a phenomenon of interest [13,14] and able to deeply reflect and narrate multidimensional experiences. In IPA research, it is recommended to keep the sample size small to focus on the quality of the interview process rather than on the quantity [12]. Ten women of the 118 that were participating in a mixed methods study that addressed the impact of Hurricane Maria and its aftermath among mothers and infants who were exposed to maternal stress related to a major catastrophe and long aftermath were invited to be part of the qualitative study reported in this manuscript. The first ten women who agree to share their lived experience as pregnant women during the Hurricane Maria in an in-depth interview were included. In the larger study maternal stress, infant outcomes, and resilience factors, were further explored and will be reported elsewhere. To be part of the qualitative part of the study they had to be 21 years or older, having been pregnant during Hurricane Maria, and willing to share their life experiences with the researcher in a face-to-face interview. The study had the approval of the Institutional Review approval from the University of Puerto Rico, Medical Sciences Campus (UPR-MSC).

A panel of experts (health educator, physician, and nurse) developed an interview guide using the most recent literature about the topic as reference. The question guide sought to elicit responses from the participants about their lived experiences during and after the hurricane, with emphasis on how they experienced access to care and social and family support.

Ten women who were pregnant at the time of Hurricane Maria participated in the interview. The interviews were conducted from 11 September 2019 to 29 January 2020, in a non-threatening environment at the Maternal and Infants Study Center at the UPR-MSC, which offers comprehensive health services to pregnant women. The interviews were conducted in Spanish, the native language of the study participants and the researchers, by the first author (GSS). GSS is a Hispanic female researcher with an educational background in public health and has worked with vulnerable populations such as sex workers, drug users, youth, and people living with an HIV diagnosis [15,16,17,18]. She has conducted and been involved in various qualitative research studies and had has formal and informal training in conducting qualitative interviews and focus groups. GSS did not know the study participants beforehand.

Each participant received a monetary incentive to cover transportation and other expenses associated with study participation. The interviews were audio recorded and transcribed and sanitized by two of the authors (HPM and SRL). A research assistant knowledgeable about Puerto Rico culture and proficient in English and Spanish, translated all the interviews. The original Spanish text was used to conduct the qualitative analysis to keep the trustworthiness of the process.

### Data Analysis

Following the steps proposed by Smith [12], transcripts were examined individually. Ensuring that the participants were the focus of the analysis, the researchers read and re-read each of the transcripts and initial observations were documented. In addition to the first author, transcripts were read and coded by two of the authors of this paper (HPM and SRL). Passages from the interviews were then discussed with other team members in relation to situation and meaning construction, as suggested by the IPA approach. Interrelations, connections, and patterns were explored using participants’ lived experiences, and narratives, establishing patterns across cases. Participants’ direct quotes under a fictional name are included to support the study findings.

## 3. Results

Ten women participated in the in-depth interviews. The mean age of the study participants was 30 years. Five of them were single, and the rest were married at the time of the interview. All of the study participants completed high school, and four of them had a college degree. Seven of them had a monthly income of less than $1000, while the rest had a monthly income between $1001 and $2000, and the majority of them had only one child.

Five major themes emerged from the participants’ lived experiences that help us understand the essence of being pregnant during Hurricane Maria in Puerto Rico. The themes are: (1) the meaning of Hurricane Maria, (2) fear of losing their pregnancy, (3) the dual burden of protecting themselves and their unborn baby, (4) disruption in health care, and (5) coping mechanisms.

### 3.1. Meaning of Hurricane Maria

For most of our participants, Hurricane Maria had a negative meaning, expressed as something “hard, catastrophic, indescribable, difficult experience.” Despite the negative feelings, most participants seemed to have a transformative experience. It was further described as a learning experience that made them stronger. On many occasions the transformation had religious and spiritual connotations. The following quotes illustrate the transformation.

*“... Is that it was very, very, very shocking. It was a big impact and truthfully is something that stays with you your whole life.” (Estela, 34 years old, mother of one).*

*“An experience that will not be forgotten… because it touched everyone, not only for what one lost, but also one learned to be human because I was pregnant, but I also learned to help others. We were more human. I learned to give... to share what I had with others.” (Carmen, 31 years old, mother of one)*

*“I learned a lot of things, you know…I learned to…that even without light or water I can survive and share with others... I learned to believe in God, that I did not…not go in a rush… I give out of my good heart, that God sees, don’t expect anything in return.” (Brenda, 45 years old, mother of three).*

### 3.2. Fear

A recurrent theme among the study participants was fear. They expressed fear of having the baby during the hurricane, fear of not having access to healthcare services, and fear of having negative outcomes including a premature birth. As one of our study participants narrated:

*“I was afraid, for the pregnancy, I was very afraid. And in the middle of that occurrence (hurricane strike), I was trying to stay calm, as I was pregnant, and they get affected also, even if they are in the belly. I was afraid to lose the baby.” (Gretel, 36 years old, mother of two).*

The disruption in health care was something that concerned most of our study participants. This is what a study participant narrated:

[talking about her concerns of needing healthcare services] *“Wow, I fear that something catastrophic happens and not having the tools or an adequate place to go, in my state [pregnancy], to receive medical care, etc.” (Soraya, 31 years old, mother of one).*

### 3.3. Dual Burden

The dual burden of protecting themselves and their unborn baby was evident in most of the participants’ experiences. It seemed to be increased by the circumstances that arise during a natural disaster. Most of the study participants narrated how they convinced themselves to be calm and cope with the situation peacefully to protect their pregnancy. While the hurricane struck the Island, they assumed the role of “savior” of their unborn baby.

*“I said: no, I have to keep calm, I know the situation is not easy, this has been extremely hard, this has impacted us, this has shocked us very hard, but I have a life inside of me that I have to take care of and save.” (Estela, 34 years old, mother of one).*

### 3.4. Health Care

Due to the unstable situation on the Island, most of the participants anticipated disruption in health care. However, most of them reported receiving the care they needed, even though they acknowledged some delays, changes in locations, and different points of access. Their overall evaluation of the healthcare system was positive.

*“The medical services here were the most excellent. Truly that, for me, I say: “if at any moment God blesses me with another baby, I would come back here for the care.” Because truthfully, everything, the attention, the doctors, the nurses, the facilities, truly excellent.” (Estela, 34 years old, mother of one).*

### 3.5. Coping Mechanisms

The strategies used by the study participants to cope with the unexpected emotional and physical barriers associated with the perceived uncertainty of their pregnancy outcome were getting distracted with family members and helping others, e.g., neighbors. Their intrinsic motivation was their babies: *“I moved forward for her [baby], because she needed me.” (Victoria, 24 years old, mother of one).*

In addition, the support of friends and family members was crucial. The closeness of family members, especially mothers was welcomed by the pregnant women.

*“Well, my family, my mom, my aunt when she’d bring things to eat at first. She would always come by every week, she’d never stay, but she would get there, would bring whatever food and bottled water and leave.” (Victoria, 24 years old, mother of one).*

## 4. Discussion

While Hurricane Maria spared no one, pregnant women faced unique and extraordinary challenges. All the participants included in this study revealed the meaning and impact of Hurricane Maria on their lives. During this challenging period, they felt fear of their baby’s health and disruptions in maternal care, which led them to feel a double burden and consequently use coping mechanisms to help manage their emotions. Most of them indicated that the hurricane was a “hard and difficult experience”, but they also described it as “transformative” and explained how it made them stronger. In addition, fear was a recurrent theme during the interviews; most women feared having a premature birth and worried about not having access to healthcare services. In a study that explored women’s experiences with pregnancy during Hurricane Katrina, most women interviewed were worried about not carrying the baby to term and the impact of stress post-Katrina would have on their babies [19]. In addition to the uncertain status of their prenatal care and delivery, the women also mentioned they feared losing their homes and jobs [19]. In a second study that observed the mental health and worries of pregnant women living through disaster recovery after Hurricane Katrina, women indicated concerns about their health, health of the developing baby and the birthing process [9]. Furthermore, some women identified the death of the baby’s father as a worry [9]. In our study, expecting mothers expressed concerns mostly related to their own health during pregnancy and that of their unborn child.

Regarding the fear of healthcare services accessibility, natural disasters such as Hurricane Maria may lead to an interruption of maternal care and limited access to clean water and safe food, which consequently poses threats to women’s health [20]. Access to adequate perinatal care following a natural disaster can be a challenge for new and expecting mothers. Women are more likely than men to receive inadequate health care following a natural disaster and the consequences are more severe for pregnant women due to the needs of the developing fetus [21,22,23]. A study that observed the impact of Hurricane Irma and Maria on Maternal and Child Research Programs explains how access to continued health care was a major issue in the wake of both hurricanes. One aspect was securing transportation to pharmacies and clinics due to loss of public transit. Further, many pharmacies were closed due to lack of electricity and those pharmacies that were open usually lacked internet access to verify prescriptions [21]. Despite that, all our participants received the care they needed; in the advent of Hurricane Maria, access to health was one of the main concerns of the participants of our study.

Additionally, most participants felt a dual burden of protecting themselves and their unborn baby; the women expressed the necessity to stay calm and cope with the situation because they wanted to focus on their health and well-being and their unborn child. A study concluded that some pregnant women managed the stress by gaining the perspective that they needed to be grounded for their health and their babies; one woman stated: “I knew that I had to be strong for him [my son]. He was the most important thing in my life at that point…If I [was] stressed out, I wasn’t going to be producing milk [19].” In the previously mentioned study about women’s mental health after Hurricane Katrina, aside from the women’s anxiety about their pregnancy, they were also concerned about the mental health of their family members and themselves and the effect it may have on their baby [9].

Regarding the coping mechanisms, participants in our study stated that interactions with family, friends, and neighbors helped them to manage their psychological stress. In addition, their babies were their intrinsic motivation. Latina women need to have their family members close by, especially in times when fear grips them. They feel secure knowing they have the support of their loved ones during and after their pregnancy [9]. A previous study that focused on pregnant women exposed to Hurricane Katrina concluded that different coping mechanisms may mitigate the effect of major stressors [24].

Natural disasters such as Hurricane Maria can have long-term physical and psychological health consequences particularly in vulnerable populations. As previously mentioned, pregnant women and infants have unique health concerns, especially after a natural disaster, and are considered among the most vulnerable [5,20]. Consequently, the identification of specific hurricane-related experiences, situations and stressors would be valuable for emergency response planning and potentially minimize them in future events [25]. Literature on the impact of expectant mothers during and after a catastrophic hurricane is limited, but studies have shown that women are at high risk of adverse effects during pregnancy [20]. Women may develop physical and mental complications associated with stress such as miscarriage, fetal death, depression, or other adverse outcomes. Depression and post-traumatic stress disorder (PTSD) are common effects after natural disasters [6]. In fact, depression is one of the most common psychological symptoms among pregnant women, regardless of their situation [26]. We must be aware of the needs of this population and be prepared since the Caribbean is vulnerable during hurricane season. In collaboration with the department of health, education campaigns and the distribution of information sheets that include details on health care in women when they are pregnant are recommended [20].

Current literature has not thoroughly explored women’s perception of disaster during pregnancy. King, Cao-Lei, and Lautarescu concentrated on maternal prenatal stress on infant outcomes [27,28,29]. Understanding the impact of disaster on pregnant women is essential to implement effective emergency preparedness plans and provide resources that will effectively allow people to cope with loss and stress while maintaining public health [19]. Our findings underscore the need to incorporate emotional support in the preparedness and response plan for pregnant women. There is a need to develop roadmaps to access care, considering the different scenarios of disruption in healthcare during hurricanes. The time has arrived for gathering more information related to identifying mechanisms, predicting risk, and developing stress-reducing and resilience-building interventions to improve pregnancy outcomes during natural events [30].

Some study limitations include the time between the hurricane and the interviews, women’s stages of pregnancy (since it may affect the results), and if the participants have a prior history of a mental health condition. Study participants were interviewed almost two years after the hurricane, and they might have experienced recall bias. However, as qualitative researchers, we were interested in what they recalled of the event since that is what has shaped their lives and has made the most significant impact on them.

Another study limitation was the small sample size. However, this is a qualitative study, and the aim was to understand participants’ lived experiences and not generalize the study findings. In future studies, it will be worth exploring depression and PTSD factors.

## 5. Conclusions

The interviews provided a wealth of firsthand information and testimonials of a group of women experiencing a natural disaster while pregnant. The participants were able to describe and reflect on their physical, emotional, and health care needs. These interviews provided evidence that allowed us to contrast and compare the experiences of pregnant women within the context of a natural disaster.

The findings underscore the need to incorporate emotional support in the preparedness and response plans for pregnant women. The bond and responsibility felt towards the unborn baby should be preeminently considered in any intervention. There is a need to develop roadmaps to access care, considering the different scenarios of disruption in healthcare during hurricanes. Families and communities were an integral part of the coping mechanisms described by pregnant women. Hence, educating, empowering, and incorporating families and communities is vital in disaster preparedness and response efforts.
